# DMSO‐ and Serum‐Free Cryopreservation of Wharton's Jelly Tissue Isolated From Human Umbilical Cord

**DOI:** 10.1002/jcb.25563

**Published:** 2016-06-23

**Authors:** Sharath Belame Shivakumar, Dinesh Bharti, Raghavendra Baregundi Subbarao, Si‐Jung Jang, Ji‐Sung Park, Imran Ullah, Ji‐Kwon Park, June‐Ho Byun, Bong‐Wook Park, Gyu‐Jin Rho

**Affiliations:** ^1^ Department of Theriogenology and BiotechnologyCollege of Veterinary Medicine, Gyeongsang National University501, Jinju‐daeroJinju660‐701Republic of Korea; ^2^ Department of Obstetrics and GynecologySchool of MedicineGyeongsang National UniversityJinju660‐702Republic of Korea; ^3^ Department of Oral and Maxillofacial SurgerySchool of MedicineGyeongsang National UniversityJinju660‐702Republic of Korea; ^4^ Research Institute of Life SciencesGyeongsang National University501, Jinju‐daeroJinju660‐701Republic of Korea

**Keywords:** CELL SURVIVAL RATE, COCKTAIL SOLUTION, CRYOPROTECTANTS, FREEZING, WHARTON'S JELLY MESENCHYMAL STEM CELLS, WHARTON'S JELLY TISSUE

## Abstract

The facile nature of mesenchymal stem cell (MSC) acquisition in relatively large numbers has made Wharton's jelly (WJ) tissue an alternative source of MSCs for regenerative medicine. However, freezing of such tissue using dimethyl sulfoxide (DMSO) for future use impedes its clinical utility. In this study, we compared the effect of two different cryoprotectants (DMSO and cocktail solution) on post‐thaw cell behavior upon freezing of WJ tissue following two different freezing protocols (Conventional [−1°C/min] and programmed). The programmed method showed higher cell survival rate compared to conventional method of freezing. Further, cocktail solution showed better cryoprotection than DMSO. Post‐thaw growth characteristics and stem cell behavior of Wharton's jelly mesenchymal stem cells (WJMSCs) from WJ tissue cryopreserved with a cocktail solution in conjunction with programmed method (Prog‐Cock) were comparable with WJMSCs from fresh WJ tissue. They preserved their expression of surface markers, pluripotent factors, and successfully differentiated in vitro into osteocytes, adipocytes, chondrocytes, and hepatocytes. They also produced lesser annexin‐V‐positive cells compared to cells from WJ tissue stored using cocktail solution in conjunction with the conventional method (Conv‐Cock). Real‐time PCR and Western blot analysis of post‐thaw WJMSCs from Conv‐Cock group showed significantly increased expression of pro‐apoptotic factors (BAX, p53, and p21) and reduced expression of anti‐apoptotic factor (BCL2) compared to WJMSCs from the fresh and Prog‐Cock group. Therefore, we conclude that freezing of fresh WJ tissue using cocktail solution in conjunction with programmed freezing method allows for an efficient WJ tissue banking for future MSC‐based regenerative therapies. J. Cell. Biochem. 117: 2397–2412, 2016. © 2016 The Authors. *Journal of Cellular Biochemistry* published by Wiley Periodicals, Inc.

Mesenchymal stem cells (MSCs) are considered to be adult stem cells, having a high capacity for self‐renewal while maintaining their multipotency. After initial identification of MSCs in the bone marrow, their existence also extends to adult tissues and organs in which they form a supportive structure and maintain the residing functional cells of that tissue. Nevertheless, the existence of MSCs in these organs not only contributes to cell turnover but also responds to tissue damage. They exert in vivo anti‐inflammatory and immunomodulatory effects [Le Blanc, 2003; Caplan and Dennis, 2006] with minimal immunoreactivity [Tse et al., 2003]. Due to these properties, many clinical trials have been initiated for regenerative treatment of various human disorders using MSCs [Singer and Caplan, 2011]. However, the success of regenerative treatments relies on the requirement of a large number of cells which makes many tissue sources incongruous because of difficulty in acquisition of cells due to invasive procedures. Though bone marrow MSCs are considered as gold standard for the use of adult MSCs, they pose several disadvantages such as invasive and painful collection procedure, occurs in low frequency at a rate of 0.001–0.01% [Castro‐Malaspina et al., 1980], and their quality also varies according to the age of the donor. Further, due to their low frequency in bone marrow aspirates, they require extensive in vitro expansion to be used as clinical doses for patients. This may further enhance the risk of culture induced epigenetic changes [Redaelli et al., 2012] as well as microbial contaminations [Gong et al., 2012]. These major drawbacks recently gain the attention of many investigators to explore alternative sources of MSCs with least invasive collection procedures.

In this context, umbilical cord, a biomedical waste serves as an alternative source of MSCs. The facile nature of MSC acquisition in relatively large numbers from different tissue compartments of the umbilical cord with non‐invasive procedure makes it a promising MSC source for regenerative medicine at clinical settings. However, MSCs present in different micro‐anatomical regions of umbilical cord differs in their phenotypic and differentiation profiles [Subramanian et al., 2015]. MSCs interspersed in the umbilical cords Wharton's jelly (WJ) tissue are considered to be the better candidates over those from other regions of the umbilical cord in terms of their clinical utility [Subramanian et al., 2015]. WJMSCs are found to be associated with higher proliferation rates and lower immunogenicity [Fong et al., 2011] and possess both mesenchymal and embryonic stem cell markers having prolonged self‐renewal and broader differentiation ability with non‐tumorigenic property [Fong et al., 2010].

In the current practice of biomedical research, cryopreservation of oocytes and embryos has made a tremendous growth that hundreds of thousands of domestic animals and laboratory animals are produced from frozen embryos [Wiles and Taft, 2010]. As MSC‐based regenerative therapies became more promising, an efficient cryopreservation and biobanking have also become progressively important [Mason and Manzotti, 2010]. Nevertheless, a large number of frozen MSCs stored in compliance with current good manufacturing practice (cGMP) will be required for clinical applications. Therefore, developing an effective technique for the cryopreservation of MSCs using cGMP‐grade reagents that are free of both animal serum proteins and toxic chemicals could increase the usefulness of these cells in tissue engineering and regenerative medicine. The use of xenogeneic animal serum in either cultivation or cryopreservation of cells impede clinical utility of MSCs as it is directly linked to the detection of anti‐FBS antibodies in patients receiving cell infusions or transplants [Sundin et al., 2007]. Moreover, cell preparations in the presence of animal serum are under increased scrutiny by regulatory authorities. Therefore, complete elimination of animal serum from being used in either cultivation or a cryopreservation protocol is the best approach to avoid possible occurrence of post‐transplantation complications. In addition, the use of conventional dimethyl sulfoxide (DMSO) in cryopreservation exerts undesirable effects on cells/tissue and even cause post‐transplantation complications [Ruiz‐Delgado et al., 2009; Yong et al., 2015]. Hence, there is a growing concern to develop both animal serum and DMSO‐free cryoprotectants, and this could be achieved probably by using different polymers either alone or in combination as cryoprotectants.

When biobanking is considered, isolating WJMSCs can be laborious, time‐consuming, and expensive, especially maintaining in compliance with cGMP for clinical use of these cells. Therefore, an ideal option to store WJMSCs which are not immediately needed is the cryopreservation of umbilical cord tissue as a whole with minimal manipulation immediately after receiving from clinics. Indeed, the success of clinical cryopreservation of human amniotic membranes [Hennerbichler et al., 2007; Parolini et al., 2008] served as a promising step towards the development of banking strategies for umbilical cord tissue by using optimal cryopreservation techniques. Although many studies found that freezing umbilical cord tissue fragments using optimal cryopreservation techniques enables their long‐term preservation, but only few considered the revitalized capacity of frozen/thaw tissue fragments in terms of cell recovery and differentiation potential of cells isolated from post‐thaw tissue [Choudhery et al., 2013a; Badowski et al., 2014; Chatzistamatiou et al., 2014; Roy et al., 2014; Shimazu et al., 2015].

Recently, it has been reported that freezing particular micro‐anatomical region of the umbilical cord has more advantage than freezing the entire umbilical cord fragments [Fong et al., 2016]. Indeed, the later poses several drawbacks such as heterogeneity in cell population and varied differentiation ability along the desired lineage as cells originate from the different compartments of the umbilical cord. Moreover, the cryoprotectants may not penetrate and preserve the interior parts of the frozen tissue resulting in genetic and behavioral changes of the cells [Fong et al., 2016]. Therefore, in the present study, we have evaluated freezing of Wharton's jelly tissue and post‐thaw behavior of WJMSCs following an optimal cryopreservation protocol using programmed slow freezing with a modified cryoprotectant, which we have earlier successfully used for cryopreserving human dental follicle tissue [Park et al., 2014].

## MATERIALS AND METHODS

### CHEMICALS AND MEDIA

Unless otherwise specified, all chemicals and media were purchased from Sigma (St. Louis, MO) and Gibco (Life Technologies, Burlington, ON, Canada), respectively.

### HARVESTING AND FREEZING OF FRESH WHARTON'S JELLY (WJ) TISSUE

After obtaining the informed consent under approved medical guidelines set by the GNUH IRB‐2012‐09‐004, human umbilical cords (n = 5) from both sexes were obtained from full‐term births undergoing either caesarean section or normal vaginal delivery. The umbilical cords (UC) were collected in sterile containers containing Dulbecco's phosphate‐buffered saline (D‐PBS) and transferred to the laboratory on ice within 2 h.

The UC was approximately cut into 2 cm pieces and thoroughly washed with D‐PBS containing 1% penicillin‐streptomycin (10,000 IU and 10,000 µg/ml, respectively; Pen‐Strep, Gibco) to remove adherent blood. The UC pieces were then cut open lengthwise with sterile forceps and curved scissors (Solco Biomedical™, Pyeongtaek, Korea). After excising both arteries and vein, the pure gelatinous WJ tissue was separated and transferred to cryovials (Thermoscientific, Roskilde, Denmark) for freezing. The experimental groups were divided into fresh (Control), conventional method with 10% DMSO diluted with advanced Dulbecco's modified Eagle's medium (ADMEM) supplemented with 10% fetal bovine serum (FBS) (Conv‐DMSO), programmed method with 10% DMSO diluted with advanced Dulbecco's modified Eagle's medium (ADMEM) supplemented with 10% fetal bovine serum (FBS) (Prog‐DMSO), conventional method with cocktail solution consisting of 0.05 M glucose, 0.05 M sucrose, and 1.5 M ethylene glycol in PBS (Conv‐Cock), and programmed method with cocktail solution consisting of 0.05 M glucose, 0.05 M sucrose, and 1.5 M ethylene glycol in PBS (Prog‐Cock). All experimental groups contain approximately the same amount of WJ tissue in each replicate. Two different freezing methods were followed. In the conventional method (Conv), WJ tissue in 1.8 ml cryovials containing 1 ml respective cryoprotectants was cooled at approximately −1°C/min from 25°C to −80°C in a freezing container (Nalgene, Rochester, NY), and then plunged directly into liquid nitrogen (−196°C) for at least 3 months. In the programmed method (Prog), WJ tissue in 1.8 ml cryovials containing 1 ml respective cryoprotectants was cooled at a pre‐set freezing rate in a programmable controlled‐rate freezer (Kryo 360, Planer Ltd, Middlesex, UK). Briefly, tissues were equilibrated for 30 min at 1°C, then cooled following the programmed protocol in order: 1°C to −9°C at a rate of −2°C/min; then −9°C to −9.1°C and held for 5 min; then −9.1°C to −40°C at a rate of −0.3°C/min; then −40°C to −140°C at a rate of −10°C/min. Then the cryovials were immediately plunged into liquid nitrogen (−196°C) and stored for at least 3 months.

### THAWING, ISOLATION, AND CULTURE OF WJMSCs

After 90 days of storage in liquid nitrogen, the cryopreserved WJ tissues were thawed by immersing in a circulating water bath at 37°C and were thoroughly washed twice with ADMEM supplemented with 10% FBS and 1% Pen‐Strep by centrifugation at 500*g* for 5 min in order to remove cryoprotectants. WJMSCs were isolated as previously described [Chao et al., 2008] with minor modifications. Briefly, the WJ tissues from all groups were minced and digested with DPBS containing 1 mg/ml collagenase type I at 37°C for 15 min with gentle agitation to loosen the gelatinous mesenchymal matrix to dislodge the interspersed MSCs. The digested tissue was then sequentially passed through 100 µm and 40 µm nylon cell strainers (BD Falcon, MA) in order to obtain a single cell suspension after enzyme being inactivated by adding ADMEM containing 30% FBS. The cell suspension was then centrifuged at 500*g* for 5 min and the pellet was reconstituted and cultured in ADMEM supplemented with 10% FBS at 37°C in a humidified atmosphere of 5% CO_2_ in air by changing the culture medium for every 3 days. Once cells became confluent (70%), they were trypsinized using 0.25% trypsin‐ethylenediaminetetraacetic acid (EDTA) solution and further expanded. WJMSCs isolated from WJ tissue without undergoing the procedure of cryopreservation was herein referred to as Fresh or Control group. In the present study passage 3 WJMSCs under each experimental group were used in all the experimentation unless otherwise specified.

### POST‐THAW MORPHOLOGY OF WJMSCs

Morphology of WJMSCs was analyzed under a light microscope at primary culture and upon passaging in all the experimental groups. Images were taken at 100× magnification with Nikon DIAPHOT 300, Japan.

### CELL SURVIVAL, CELL RECOVERY, AND GROWTH CHARACTERISTICS OF WJMSCs

After isolating cells from both fresh and cryopreserved groups of WJ tissue, they were stained with propidium iodide (PI) for dead cells and Hoechst 33342 for all cells as previously reported [Park et al., 2014]. The stained cells were then observed using a fluorescent microscope (Nikon Eclipse Ti‐U, Nikon Instruments, Tokyo, Japan) and the rate of cell survivability is calculated in each experimental group.

To evaluate the total number of viable cells recovered from both fresh and cryopreserved groups of WJ tissue, the isolated WJMSCs were stained with 0.4% Trypan blue (Sigma–Aldrich Corp., St. Louis, MO) for 1 min at room temperature and then the number of live cells was counted using a hemocytometer and expressed as the number of cells recovered per cm of umbilical cord.

To compare the growth characteristics of WJMSCs isolated from fresh and cryopreserved tissue, the plating efficiency, population doubling time (PDT), and saturation density were measured.

The colony forming ability (Plating efficiency) was evaluated as previously described [Choudhery et al., 2012]. Briefly, at passage 1, WJMSCs in each experimental group were seeded in triplicates in 25 cm^2^ culture flasks at 20 cells per cm^2^ and propagated in ADMEM supplemented with 10% FBS for 14 days. After 2 weeks, resulting colonies were fixed with methanol and stained with crystal violet (0.1%). Colonies with >30 cells were counted manually under a microscope. Colonies were counted by two independent observers and the plating efficiency was measured by using the formula: number of colonies counted/number of cells initially plated and then multiplied by 100.

The proliferation rate of WJMSCs was evaluated by population doubling time (PDT). Briefly, WJMSCs isolated from all the experimental groups were seeded at 2 × 10^3^ cells per well in triplicates using 24‐well culture plate. Cells were propagated for up to 14 days and the cell number was recorded for every 2 days interval. PDT was calculated using a formula, PDT = *t* (log2)/(log*N_t _*− log*N_0_*), where *t* represents the culture time, and *N_0_* and *N_t_* are the initial and final cell numbers before and after seeding, respectively.

The saturation density of WJMSCs in each experimental group was determined in triplicates as previously described [Choudhery et al., 2013b]. Briefly, at passage 1, cells were trypsinized, counted and replated in 25 cm^2^ culture flasks at a final concentration of 1000 cells per square centimeter. The cells were observed daily under a microscope until becoming confluent. The cells were counted every other day using a hemocytometer until the cessation of further increase in cell number.

### FLOW CYTOMETRY

WJMSCs were analyzed for the expression of surface antigens and DNA content using flow cytometer (BD FACS Calibur; Becton Dickinson, NJ) in triplicates from three independent experiments. For phenotyping of surface antigens, WJMSCs were harvested using 0.25% Trypsin‐EDTA and fixed in 3.7% formaldehyde solution. The cells were then washed twice with DPBS and labelled (1 × 10^5^ cells per marker) with fluorescein isothiocyanate‐conjugated CD34 (BD Pharmingen, CA, FITC Mouse Anti‐Human CD34), CD45 (Santa Cruz Biotechnology, FITC Mouse Anti‐Human CD45), CD90 (BD Pharmingen, FITC Mouse Anti‐Human CD90) and unconjugated CD73 (Santa Cruz Biotechnologies, Mouse monoclonal), and CD105 (Santa Cruz Biotechnologies, Mouse monoclonal IgG_2a_) for 30 min. Unconjugated primary antibodies were treated with secondary FITC‐conjugated goat anti‐mouse IgG (BD Pharmingen) for 30 min in the dark. For isotype matched negative control Mouse IgG1 (BD Pharmingen) was used. A total of 10,000 labeled cells per sample were acquired and results were analyzed using cell Quest Pro software (Becton Dickinson). For evaluating DNA content, a total of 1 × 10^6^ cells/ml were fixed in 70% ethanol at 4°C for 4 h. After washing cells twice with DPBS, they were stained with 10 µg/ml PI solution for 15 min. DNA content of each cell was measured and categorized as G_0_/G_1_, S, or G_2_/M phase of the cell cycle.

### DETECTION OF RECURRENCE OF APOPTOSIS

In order to detect the possible recurrence of apoptosis due to cryoinjury in WJMSCs isolated from post‐thaw WJ tissue, a well‐established Annexin V apoptosis assay was conducted by quantitative flow cytometry using FITC Annexin V Apoptosis Detection Kit 1 (BD Pharmingen, CA). Briefly, both detached and attached cells at passage 1 were pooled, harvested by trypsinization (0.25% trypsin), washed twice with cold DPBS and resuspended cells in 1X binding buffer and stained with Annexin V‐FITC and PI for 15 min at room temperature in dark, then added additional 400 µl of 1X binding buffer and immediately analyzed by flow cytometry within 1 h. Cell viability and apoptosis/necrosis assessment were made using FACSCaliber flow cytometer (BD FACS Calibur; Becton Dickinson, NJ) using 488‐nm laser excitation and fluorescence emission at 530 nm (FL1) and >575 nm (FL3). A total of 15,000 cells per sample were acquired in triplicate from three independent experiments using cell Quest Pro software (Becton Dickinson). Linear amplification was used for forward‐ and side‐scatter measurements and logarithmic amplification was used for all the fluorescence measurements. The fluorescent dot plots have three cell populations: live (annexin V‐FITC‐negative/PI‐negative), necrotic (annexin V‐FITC‐positive/PI‐positive), and apoptotic (annexin V‐FITC‐positive/PI‐negative). Quadrant analysis was performed on the gated fluorescent dot plot to quantify the percentage of live, necrotic, and apoptotic cell populations. The quadrant positions were placed according to the non‐cryopreserved sample (Control/Fresh).

### IN VITRO MESENCHYMAL LINEAGE DIFFERENTIATION

WJMSCs from both fresh and cryopreserved groups were evaluated for their in vitro differentiation ability into osteogenic, adipogenic, and chondrogenic lineages as per the previously published protocols [Patil et al., 2014]. Briefly, cells were cultured in ADMEM supplemented with lineage‐specific constituents for 21 days by changing media for every 3 days interval. Osteogenic medium comprised of 0.1 µM dexamethasone, 50 µM ascorbate‐2‐phosphate, and 10 mM glycerol‐2‐phosphate. Osteogenesis was confirmed by alizarin red and von Kossa staining. Adipogenic medium comprised of 1 µM dexamethasone, 10 µM insulin, 100 µM indomethacin, and 500 µM isobutylmethylxanthine (IBMX). Adipogenesis was confirmed by the accumulation of lipid droplets by staining with Oil red O solution. Chondrogenesis was induced by using the commercial chondrogenic medium (StemPro^®^ Osteocyte/Chondrocyte Differentiation Basal Medium; StemPro^®^ Chondrogenesis supplement, Gibco by life technology) and differentiation was evaluated by Alcian blue and Safranin O staining.

### IN VITRO HEPATOGENIC DIFFERENTIATION

The differentiation ability of WJMSCs isolated from both fresh and cryopreserved tissues towards hepatogenic lineage was evaluated as previously described [Patil et al., 2014]. Briefly, cells after reaching 70% confluence in ADMEM supplemented with 10% FBS were further cultured with hepatocyte priming medium consisted of ADMEM supplemented with 2% FBS and 20 ng/ml recombinant human hepatocyte growth factor (HGF, R&D Systems, Inc., MN) for 7 days. Primed cells were then cultured in hepatocyte maturation medium consisted of ADMEM supplemented with 2% FBS, 10 ng/ml oncostatin M (R&D Systems, Inc.,), 10 nmol/l dexamethasone and 1% insulin‐transferrin‐selenium mix (ITS‐mix) for 15 days. Both priming and maturation media were changed on every alternative day. Control cultures were also maintained in parallel to the differentiation experiments.

### IMMUNOCYTOCHEMISTRY

Immunocytochemical staining was performed by fixing cells with 3.7% formaldehyde for 30 min and permeabilized with 0.25% Triton X‐100 for 10 min at room temperature. After blocking with 1% bovine serum albumin (BSA) in DPBS for 1 h, cells were incubated with primary antibodies, such as goat anti‐Oct‐3/4 (1:200, Santa Cruz Biotechnology, CA), rabbit anti‐Sox‐2 (1:200, Santa Cruz Biotechnology), goat anti‐Nanog (1:200, Santa Cruz Biotechnology), goat anti‐human serum albumin (ALB, 1:200, Santa Cruz Biotechnology) and goat anti‐hepatocyte nuclear factor 1‐alpha (HNF‐1α, 1:200, Santa Cruz Biotechnology) for overnight at 4°C followed by incubation with CruzFluor™ 594 conjugated donkey anti‐goat IgG (1:200, Santa Cruz Biotechnology) or donkey anti‐rabbit IgG (1:200, Santa Cruz Biotechnology) secondary antibodies for 45 min at 37°C. The nuclei of cells were counterstained with 1 µg/ml 4′,6‐diamidino‐2‐phenylindole (DAPI) for 5 min and images were taken using fluorescence microscope (Leica, Wetzlar, Germany).

### REAL TIME‐POLYMERASE CHAIN REACTION (RT‐PCR)

The expression of transcription factors, apoptosis‐related genes, and lineage‐specific marker genes was analyzed by RT‐PCR in triplicates from three independent experiments. Total RNA was isolated using the RNeasy mini kit (Qiagen, Valencia, CA) from control or induced WJMSCs from all the experimental groups. A total of 2 µg RNA was used to synthesize complementary DNA (cDNA) using Omniscript RT kit (Qiagen) with oligo‐dT primer. The reaction was carried out at 37°C for 60 min. Real‐time PCR was carried out on a Rotor gene Q (Qiagen) using Rotor Gene™ SYBR green PCR kit (Qiagen). A total of 50 ng cDNA was added to 12.5 µl SYBR Green mix, 5.5 µl RNase free water and 1 µl each of forward and reverse primers at 1 pM (final volume 25 µl). The assay was performed with initial denaturation at 95°C for 10 min, followed by 40 PCR cycles of 95°C for 10 s, 60°C for 6 s, and 72°C for 4 s, followed by a melting curve from 60°C to 95°C at 1°C/s and then cooling at 40°C for 30 s, according to the manufacturer's protocol. CT values and melting curves of each sample were analyzed using Rotor‐Gene Q series software (Qiagen). The PCR products were evaluated by 1.5% agarose gel electrophoresis and images were analyzed using zoom browser EX5.7 software (Canon). YWHAZ (Tyrosine 3‐monooxygenase/tryptophan 5‐monooxygenase activation protein, zeta polypeptide) was used as a housekeeping gene for normalization of the data. The relative level of target gene expression was calculated according to the 2^−ΔΔCT^ method. The primers used are listed in Table I.

**Table I:** List of Primers Used for the Evaluation of Pluripotent Genes, Apoptosis‐Related Genes, and Lineage‐Specific Marker Genes in cultured WJMSCs by RT‐PCR

Gene	Primer sequence	Product size (bp)	Accession no.
*OCT4*	F: AAGCAGCGACTATGCACAAC	140	NM_002701.5
	R: AGTACAGTGCAGTGAAGTGAGG		
*SOX2*	F: CACCCACAGCAAATGACAGC	120	NM_003106.3
	R: AGTCCCCCAAAAAGAAGTCCAG		
*NANOG*	F: GCAGATGCAAGAACTCTCCAAC	133	AB093576.1
	R: CTGCGTCACACCATTGCTATTC		
*BAX*	F: TCTGACGGCAACTTCAACTG	127	NM_001291428.1
	R: AGTCCAATGTCCAGCCCATG		
*BCL2*	F: GGCTGGGATGCCTTTGTG	66	BC027258.1
	R: CAGCCAGGAGAAATCAAACA		
*p53*	F: AATAGGTGTGCGTCAGAAGC	92	AB082923.1
	R: CCACAACAAAACACCAGTGC		
*p21*	F: TGGCAGTAGAGGCTATGGA	178	NM_000389.4
	R: AACAGTCCAGGCCAGTATG		
*RUNX2*	F: ATGTGTGTTTGTTTCAGCAG	199	NM_001024630.3
	R: TCCCTAAAGTCACTCGGTAT		
*OSTEONECTIN*	F: GTGCAGAGGAAACCGAAGAG	202	J03040.1
	R: AAGTGGCAGGAAGAGTCGAA		
*BMP2*	F: TAGACCTGTATCGCAGGCAC	149	NM_001200.2
	R: GGTTGTTTTCCCACTCGTTT		
*PPARγ*	F: TTGCTGTCATTATTCTCAGT	124	AB565476.1
	R: GAGGACTCAGGGTGGTTCAG		
*FABP4*	F: TGAGATTTCCTTCATACTGG	128	NM_001442.2
	R: TGGTTGATTTTCCATCCCAT		
*LPL*	F: AGACACAGCTGAGGACACTT	137	NM_000237.2
	R: GCACCCAACTCTCATACATT		
*SOX9*	F: ATGGAGCAGCGAAATCAACG	118	BC007951.2
	R: CAAAGTCCAAACAGGCAGAGAG		
*AGGRECAN*	F: GAATGGGAACCAGCCTATACC	98	NM_001135.3
	R: TCTGTACTTTCCTCTGTTGCTG		
*COLLAGEN II*	F: GAGACCTGAAACTCTGCCACC	165	NM_001844.4
	R: TGCTCCACCAGTTCTTCTTGG		
*AFP3*	F: GCCACTTGTTGCCAACTCAG	164	NM_001134.2
	R: CTGAAGCATGGCCTCCTGTT		
*ALB3*	F: CAGGCGACCATGCTTTTCAG	243	NM_000477.5
	R: TTATCGTCAGCCTTGCAGCA		
*HNF4A1*	F: GGAAGTGGCTGAGTCAGGAC	129	NM_178849.2
	R: CGGAAGCCCCTCAACTTGAT		
*YWHAZ*	F: ACGAAGCTGAAGCAGGAGAAG	111	BC108281.1
	R:TTTGTGGGACAGCATGGATG		

### WESTERN BLOTTING

Protein lysate was prepared from control or differentiated cells using RIPA buffer (Thermo Scientific, Rockford, IL) containing protease inhibitors. The concentration of protein was determined using Microplate BCA Protein Assay kit (Pierce Biotechnology, Rockford, IL) and a total of 25 µg each protein sample was separated by 12% sodium dodecyl sulfate‐polyacrylamide gel electrophoresis (SDS–PAGE, Mini Protean, BioRad, Hercules, CA) and transferred onto polyvinylidene difluoride membranes (PVDR, Millipore). Membranes were then incubated with primary antibodies such as goat anti‐Oct‐3/4 (1:200, Santa Cruz Biotechnology), rabbit anti‐Sox‐2 (1:200, Santa Cruz Biotechnology), goat anti‐Nanog (1:200, Santa Cruz Biotechnology), rabbit anti‐Bax (1:1000, Enzo, Farmingdale, NY), rabbit anti‐Bcl‐2 (1:1000, Cell Signaling Technology, Cambridge, MA), mouse anti‐p53 (1:200, Santa Cruz Biotechnology), rabbit anti‐p21 (1:200, Santa Cruz Biotechnology), goat anti‐human serum albumin (ALB, 1:200, Santa Cruz Biotechnology), goat anti‐hepatocyte nuclear factor 1‐alpha (HNF‐1α, 1:200, Santa Cruz Biotechnology), and rabbit anti‐β actin (1:1000, Cell Signaling Technology) for overnight at 4°C followed by incubation with horseradish peroxidase (HRP)‐conjugated donkey anti‐goat IgG (1:10,000, Santa Cruz Biotechnology), goat anti‐rabbit IgG (1:10,000, Santa Cruz Biotechnology), and goat anti‐mouse IgG (1:10,000, Santa Cruz Biotechnology) secondary antibodies for 1 h at room temperature. Immunoreactivity was detected by enhanced chemiluminescence (ECL; Supersignal, West Pico Chemiluminescent substrate, PIERCE, IL) and exposed to X‐ray films.

### PERIODIC ACID‐SCHIFF (PAS) STAINING

WJMSCs differentiated to hepatocytes were evaluated for their glycogen storage ability using PAS staining. Briefly, both control and differentiated cells were fixed in 3.7% formaldehyde for 30 min. Cells were then treated with oxidizing agent 1% periodic acid for 5 min at room temperature and rinsed three times with distilled water before treating with Schiff's reagent for 15 min at room temperature. Finally, cells were rinsed with distilled water for 5–10 min and counter stained with Mayer's hematoxylin for 30 s and washed with distilled water. Glycogen storage was observed under a light microscope.

### UREA ASSAY

After incubating both control and differentiated cells in culture medium supplemented with 1 mM ammonium chloride (NH_4_Cl) for 24 h, culture supernatants were collected, centrifuged at 300*g* for 5 min and urea levels were measured in 96‐well plates at 570 nm as per manufacturer's instruction manual (Abcam, Cambridge, MA). Fresh culture medium supplemented with 1 mM NH_4_Cl was used as a control.

### LOW‐DENSITY LIPOPROTEIN (LDL) UPTAKE ASSAY

The uptake of LDL by hepatocyte differentiated WJMSCs was evaluated using Dil AcLDL (Low‐Density Lipoprotein from Human Plasma, Acetylated, Dil complex, Thermo Fisher Scientific, MA). Briefly, cells were incubated in serum‐free DMEM‐LG supplemented with 10 µg/ml Dil AcLDL for 4 h at 37°C. Cells were then washed and visualized under a fluorescent microscope.

### STATISTICAL ANALYSIS

The statistical differences between experimental groups were analyzed by one‐way ANOVA using SPSS 21.0. For multiple comparisons, Tukey's test was performed and data were presented as a mean ± standard error of the estimate of mean value (S.E.M) for each sample measured in triplicates obtained from three independent experiments. Results were considered significant when *P* < 0.05.

## RESULTS

### SURVIVAL RATE, CELL RECOVERY, POST‐THAW MORPHOLOGY, AND GROWTH CHARACTERISTICS OF WJMSCs

**Figure 1:**
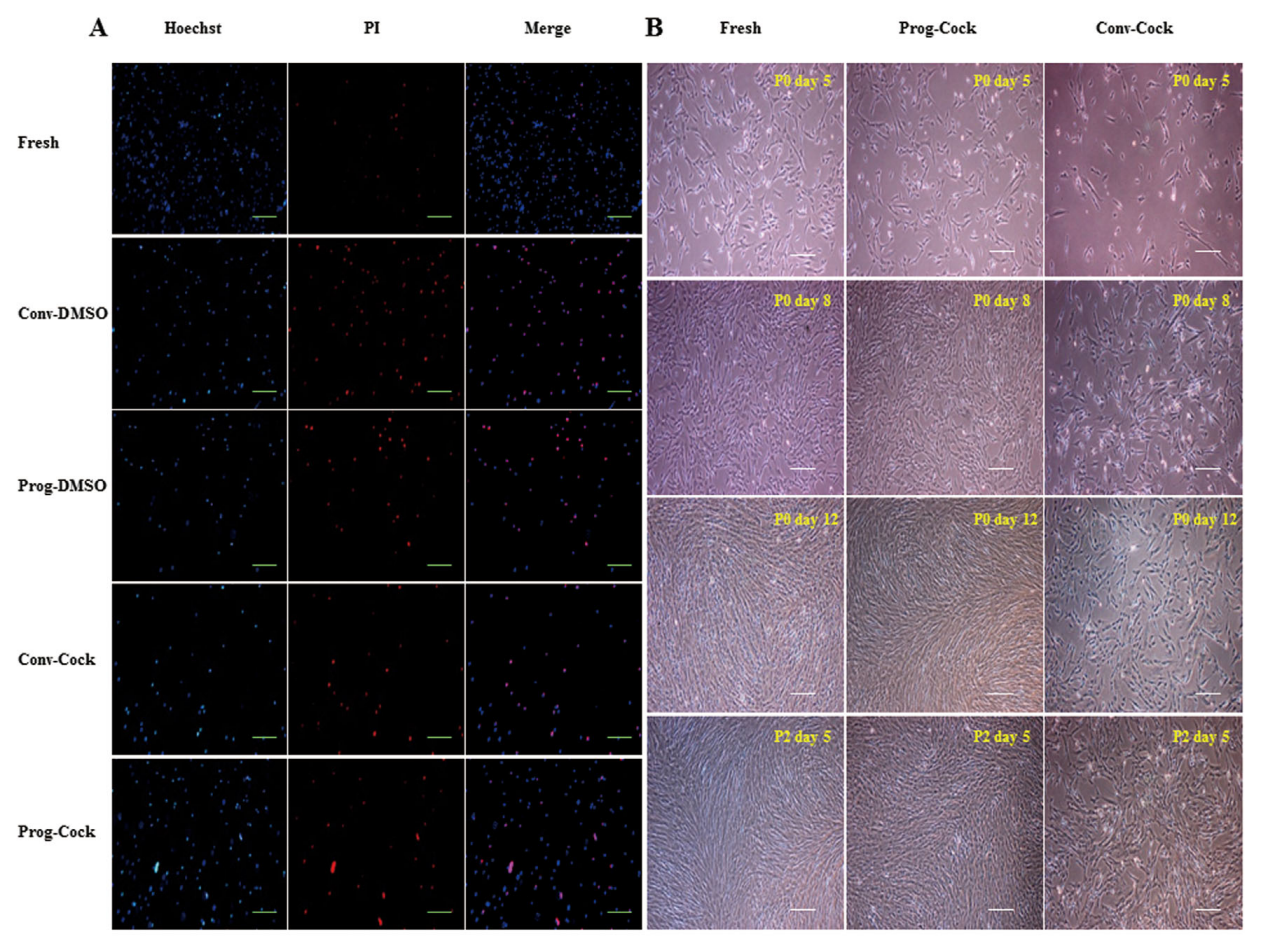
(A) Hoechst 33342 and propidium iodide (PI) staining in WJMSCs after isolating from both fresh and cryopreserved Wharton's jelly tissue; scale bar = 100 μm. (B) Phase‐contrast microscopic images showing morphology of WJMSCs from fresh, Prog‐Cock, and Conv‐Cock group; scale bar = 100 μm.

The survival rates of WJMSCs isolated from either fresh or cryopreserved WJ tissue were 93.63 ± 3.32%, 37.70 ± 4.10%, 44.60 ± 2.14%, 61.60 ± 4.57%, and 70.77 ± 2.52% in Fresh, Conv‐DMSO, Prog‐DMSO, Conv‐Cock and Prog‐Cock groups, respectively (Fig. 1A). Significantly (*P* < 0.05) higher cell survivability was achieved using the programmed method of freezing than the conventional method. Further, cocktail cryoprotectant has showed improved cryoprotection of WJ tissue than DMSO. The total number of live cells recovered per cm of umbilical cord from either fresh or cryopreserved WJ tissue following WJMSC isolation prior to culture was found to be 4.24 ± 5.3 × 10^6^, 1.52 ± 6.1 × 10^6^, 1.80 ± 3.8 × 10^6^, 2.42 ± 5.4 × 10^6^, and 3.12 ± 4.6 × 10^6^ in Fresh, Conv‐DMSO, Prog‐DMSO, Conv‐Cock, and Prog‐Cock groups, respectively. Based on these results, only the cryopreservation with a cocktail solution (Conv‐Cock and Prog‐Cock) was chosen for subsequent experiments in order to address the feasibility of complete elimination of animal serum and DMSO for WJ tissue cryopreservation.

**Figure 2:**
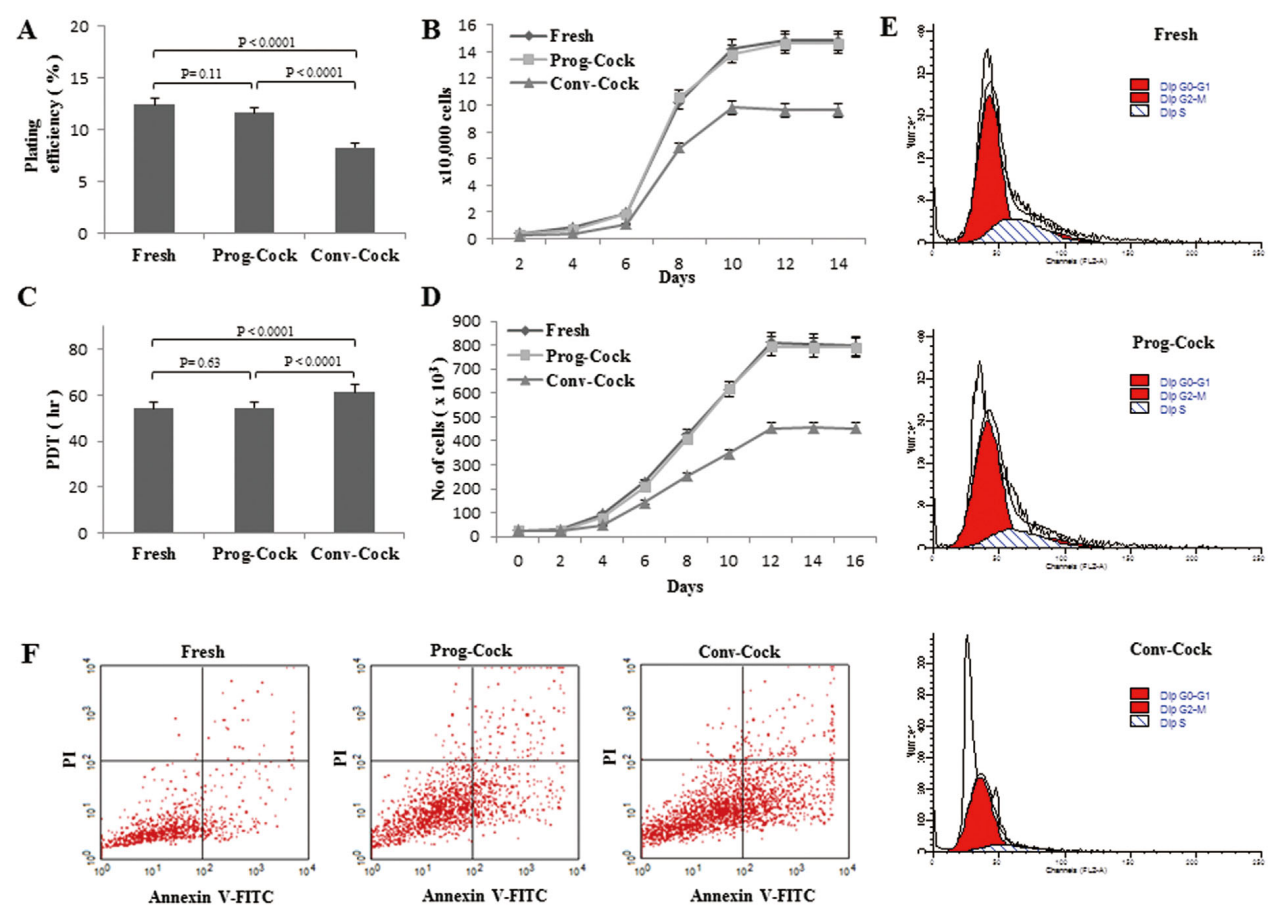
Post‐thaw growth characteristics and viability of WJMSCs in fresh, Prog‐Cock, and Conv‐Cock group. (A) Percentage of plating efficiency (colony forming ability). (B and C) Cell proliferation and population doubling time. (D) Saturation density. (E) Flowcytometric analysis of cell cycle. A total of 10,000 cells were counted for each sample in triplicates from three independent experiments. (F) Characteristic flowcytometer fluorescent dot plots showing fluorescence‐activated cell sorting analysis of post‐thaw recurrence of apoptosis due to cryoinjury in cultured WJMSCs at passage 1 as determined by annexin V staining and propidium iodide (PI) uptake. A total of 15,000 cells were counted for each sample in triplicates from three independent experiments.

WJMSCs from post‐thaw WJ tissue in primary culture have shown colonies of adherent and fibroblastic spindle‐like morphology on day 5, which became completely confluent by day 12. However, cells isolated from Conv‐Cock group have shown retarded growth and reduced cell clumps on day 5 (Fig. 1B). The total cell numbers on day 12 in primary culture were 5.5 ± 1.2 × 10^6^, 5.3 ± 1.8 × 10^6^, and 2.7 ± 1.4 × 10^6^ in Fresh, Prog‐Cock and Conv‐Cock group respectively. WJMSCs from both fresh and cryopreserved WJ tissues grew into colonies when grown in low numbers in 25 cm^2^ culture flasks to evaluate their plating efficiency. Cells from Conv‐Cock have shown reduced colony forming ability compared to that of Fresh and Prog‐Cock group of WJMSCs (Fig. 2A). Analysis of PDT showed that the proliferative capacity was comparable between cells isolated from WJ tissue of fresh and Prog‐Cock group, whereas, cells from Conv‐Cock group showed significantly (*P* < 0.05) reduced proliferative capacity. Doubling time was found to be 54.0 ± 2.4 h, 54.47 ± 3.1 h, and 61.36 ± 1.9 h for Fresh, Prog‐Cock, and Conv‐Cock group, respectively (Fig. 2B and C). The saturation density of WJMSCs in all the experimental groups reached by day 12 of culture with the number of cells at saturation density were 812,000 ± 14.3, 794,000 ± 12.1, and 452,000 ± 18.2 in Fresh, Prog‐Cock, and Conv‐Cock group, respectively (Fig. 2D). Conv‐Cock group has shown a significant difference (*P* < 0.05) in all the phases of cell cycle in comparison to that in Fresh and Prog‐Cock groups as analyzed by FACS (Fig. 2E). The proportion of cells in G_0_/G_1_ phase was 66.31 ± 1.9%, 69.26 ± 2.7%, and 82.77 ± 1.4%, whereas in S phase was 23.7 ± 2.8%, 19.73 ± 4.1%, and 14.93 ± 2.2% and those in G2/M phase was 9.99 ± 1.1%, 11.01 ± 2.6%, and 2.3 ± 4.6% in Fresh, Prog‐Cock, and Conv‐Cock group, respectively.

### RECURRENCE OF APOPTOSIS

The possible recurrence of post‐thaw apoptosis due to cryoinjury was evaluated using Annexin V‐FITC assay. The results showed significantly (*P* < 0.05) greater apoptosis signals for the cells from the post‐thaw Conv‐Cock group compared to cells from post‐thaw Fresh and Prog‐Cock groups (Fig. 2F). The proportion of viable cells was 96.26 ± 1.23%, 92.58 ± 1.87%, and 81.57 ± 0.97% whereas the cells undergoing apoptosis was 2.92 ± 1.81%, 4.56 ± 0.52%, and 15.33 ± 1.11%, and the cells in their late stage of apoptosis or dead was 0.74 ± 1.22%, 2.26 ± 0.75%, and 2.38 ± 0.63% in Fresh, Prog‐Cock, and Conv‐Cock group, respectively.

### EXPRESSION OF CELL SURFACE ANTIGENS

**Figure 3:**
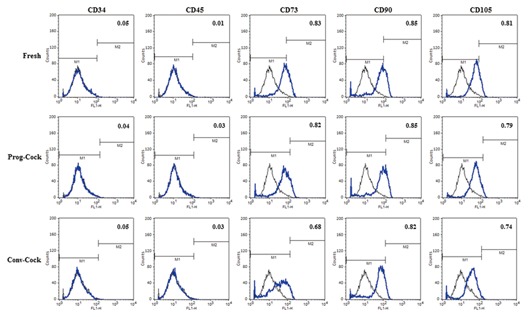
Flowcytometric analysis of the expression of surface markers by WJMSCs under fresh, Prog‐Cock, and Conv‐Cock group. WJMSCs were negative for CD34 and CD45 and positive for CD73, CD90, and CD105 expression. Significant differences were considered when *P* < 0.05.

Flow cytometric analysis of WJMSCs from both fresh and cryopreserved groups showed that they were negative for CD34 and CD45 whilst positive for CD73, CD90, and CD105 with no significant differences in CD marker expression between cells isolated from fresh and cryopreserved WJ tissue. However, cells from Conv‐Cock group showed significantly (*P* < 0.05) reduced expression of CD73 (Fig. 3).

### IN VITRO MESENCHYMAL LINEAGE DIFFERENTIATION

**Figure 4:**
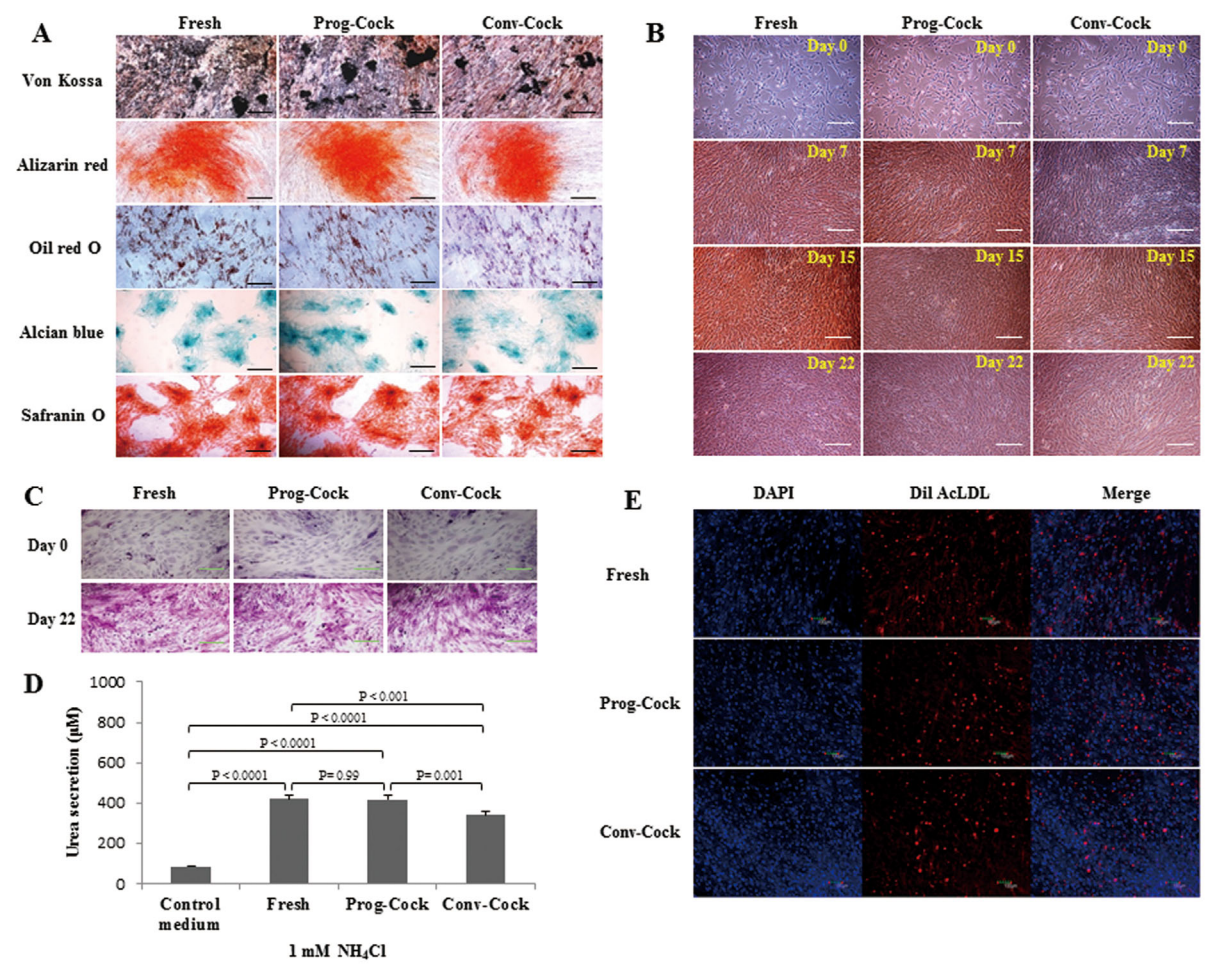
(A) In vitro mesenchymal lineage differentiation potential of WJMSCs into osteocytes (Von Kossa and alizarin red), adipocytes (Oil red O), and chondrocytes (Alcian blue and safranin O); scale bar = 100 μm. (B) In vitro transdifferentiation of WJMSCs into hepatocyte‐like cells, the sequential morphological changes from day 0 through day 22 were observed under a microscope; scale bar = 100 μm. (C) Glycogen storage ability of differentiated hepatocyte‐like cells was indicated by Periodic acid‐Schiff (PAS) staining; scale bar = 100 μm. (D) Production of urea in differentiated hepatocyte‐like cells after incubation with 1 mM NH_4_Cl. Value represents the mean ± standard error of the estimate of mean value (S.E.M) of urea secretion in μM obtained in triplicates from three independent experiments. Significant differences were considered when *P *< 0.05. (E) Fluorescent images representing the ability of differentiated hepatocyte‐like cells to uptake Dil AcLDL after 4 h incubation at 37°C; scale bar = 100 μm.

WJMSCs from both fresh and cryopreserved groups upon in vitro differentiation under specific conditions using lineage‐specific differentiation medium were able to differentiate into mesenchymal lineages (osteogenic, adipogenic, and chondrogenic). The formation of mineralized nodules upon osteogenic induction was shown by Alizarin red and von Kossa staining (Fig. 4A). Differentiated cells expressed osteogenic lineage markers runt‐related transcription factor‐2 (*Runx2*), osteonectin and bone morphogenetic protein 2 (*BMP2*) (Figs. 7A and 8C). The accumulation of intracellular lipid droplets upon adipogenic induction was demonstrated by Oil red O staining (Fig. 4A) and differentiated cells expressed adipogenic lineage markers peroxisome proliferative activated receptor gamma (*PPARγ*), fatty acid binding protein 4 (*FABP4*), and lipoprotein lipase (*LPL*) (Figs. 7B and 8D). Chondrogenic differentiation was confirmed by the deposition of sulfated proteoglycans and glycosaminoglycan's as indicated by the Alcian blue and Safranin O staining respectively (Fig. 4A). Further, differentiated cells expressed chondrogenic lineage markers SRY‐Box 9 (*Sox9*), *aggrecan*, and *collagen II* (Figs. 7C and 8E).

### IN VITRO HEPATOGENIC DIFFERENTIATION

**Figure 5:**
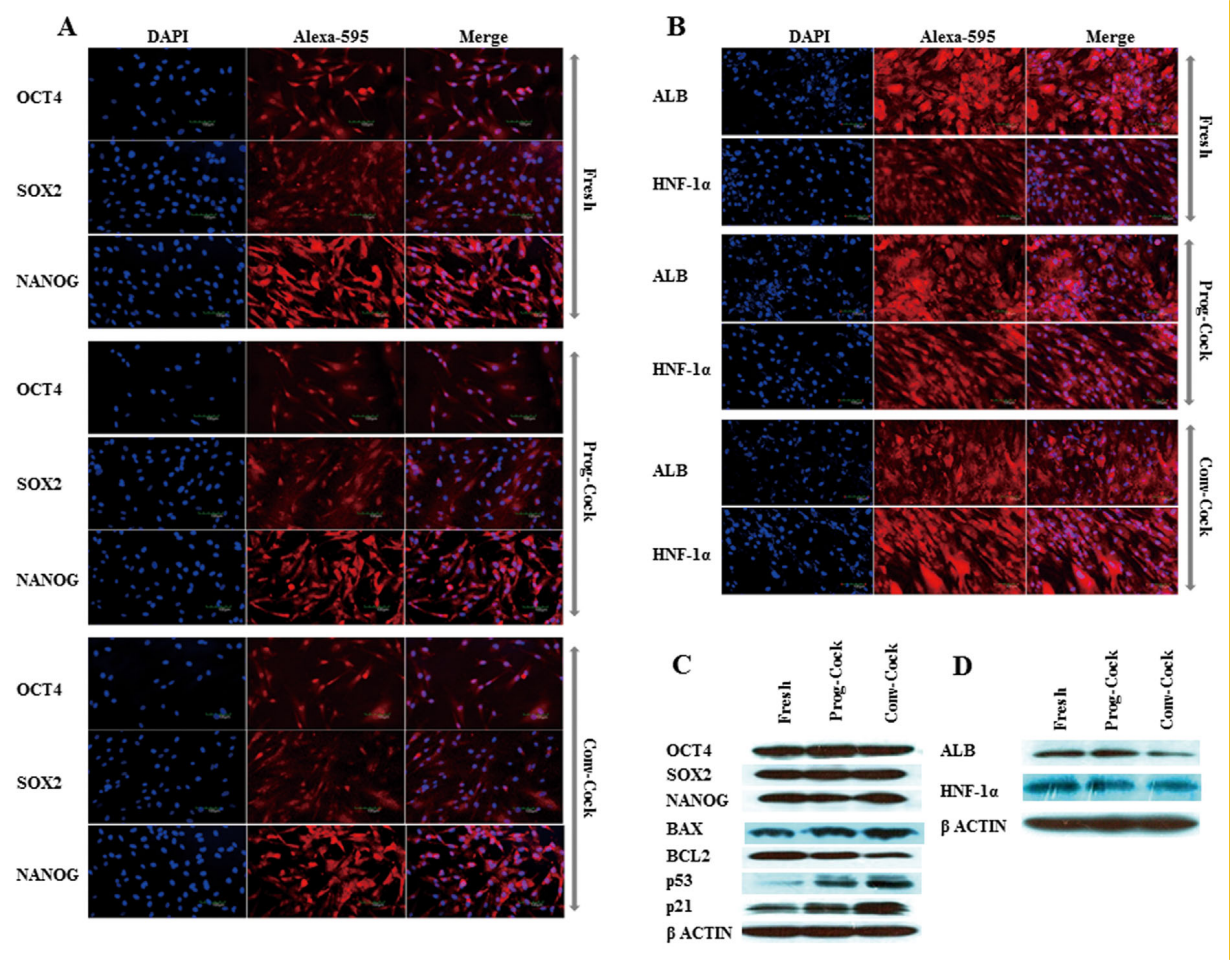
(A) Immunocytochemical analysis of OCT4, SOX2, and NANOG in WJMSCs isolated from Fresh, Prog‐Cock, and Conv‐Cock group; scale bar = 100 μm. (B) Immunocytochemical analysis of ALB and HNF‐1α in differentiated hepatocyte‐like cells; scale bar = 100 μm. (C) Western blot analysis of pluripotent‐ and apoptosis‐related proteins. (D) Western blot analysis of hepatocyte specific marker proteins.

In order to determine whether cryopreservation affects transdifferentiation ability of WJMSCs isolated from both fresh and cryopreserved WJ tissue, WJMSCs were allowed to grow until 70% confluence before hepatogenic induction. Upon induction, the fibroblast‐like morphology of WJMSCs was gradually changed to flattened shape during the priming stage of differentiation. We observed islands of adherent round or polygonal shaped cells surrounded by spindle‐shaped MSCs during the first week of maturation step. Further, change in cell morphology was more obvious during the second week of maturation step, where WJMSCs displayed hepatocyte‐like morphology in all the experimental groups (Fig. 4B). Differentiated cells expressed hepatocyte‐specific markers AFP3, ALB3, and HNF4A1 at m‐RNA and protein level although with different expression levels (Figs. 5D and 8A, B). Additionally, the immunocytochemical analysis revealed that the morphological changes observed during differentiation were sustained with the expression of ALB and HNF‐1α consistently in all the experimental groups of MSCs (Fig. 5B). The functional characteristics of HLCs were evaluated by PAS staining, LDL uptake, and urea synthesis assay. The glycogen storage capacity of differentiated WJMSCs was analyzed by PAS staining. The results showed a low level of PAS staining in undifferentiated stage (day 0) of WJMSCs in all the experimental groups. However, after exposure to the hepatogenic medium, cells displayed a significant positive staining of glycogen granules in their cytoplasm at the end of maturation (Fig. 4C). In addition, differentiated cells showed a capacity to accumulate low‐density lipoprotein (LDL) inside the cells (Fig. 4E) whereas undifferentiated cells (day 0) did not show this ability (data not shown). Finally, the ureogenesis was analyzed for the metabolic function of differentiated WJMSCs to detoxify ammonia to less toxic urea. The results showed that differentiated cells produced 4.5‐ to 4.8‐fold more urea compared to control medium (*P* < 0.05). The capacity for urea synthesis in differentiated cells was observed to be comparable in both Fresh and Prog‐Cock group. However, cells from Conv‐Cock group showed significantly (*P* < 0.05) reduced capacity for urea synthesis compared to both Fresh and Prog‐Cock group (Fig. 4D). Taken together, these data suggest that WJMSCs isolated from both fresh and cryopreserved group (Conv‐Cock and Prog‐Cock) could commit toward functional hepatocyte‐like cells upon hepatogenic induction albeit with varying capacity.

### IMMUNOCYTOCHEMISTRY

Immunocytochemistry was performed to visualize the localization of transcription factors and hepatocyte‐specific lineage markers at the protein level. The nuclear expression of Oct‐3/4, Sox‐2, and Nanog was detected in most of the cells isolated from both fresh and cryopreserved groups. However, Nanog was also occasionally observed in the cell cytoplasm (Fig. 5A). WJMSCs after differentiating into hepatocyte lineage displayed cytoplasmic expression of human serum albumin (ALB) and nuclear expression (predominant) of hepatocyte nuclear factor 1‐alpha (HNF‐1α) (Fig. 5B).

### RT‐PCR AND WESTERN BLOTTING

**Figure 6:**
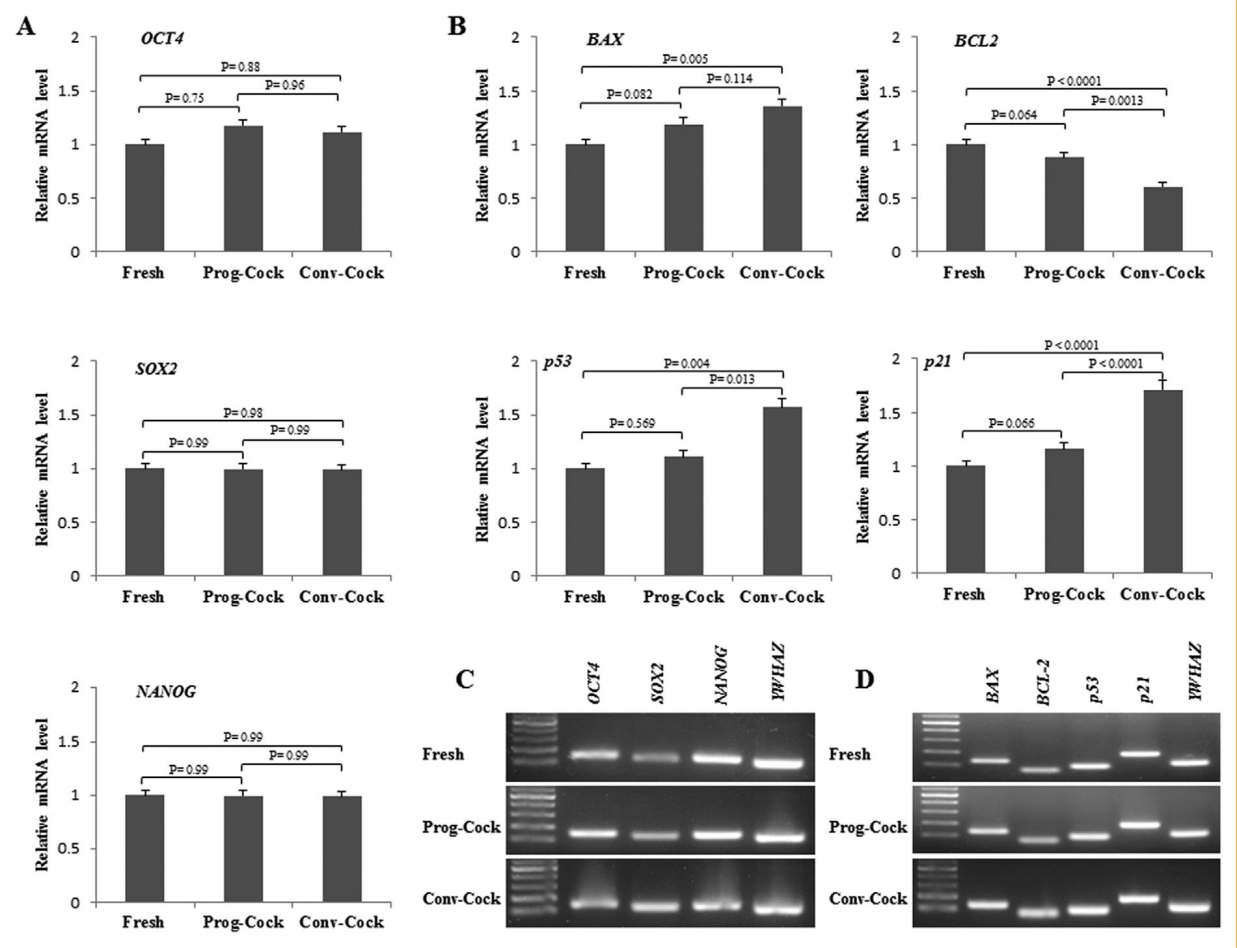
RT‐PCR analysis in cultured WJMSCs. (A) Relative m‐RNA level of pluripotent genes and (C) their product size. (B) Relative m‐RNA level of apoptosis‐related genes and (D) their product size. Significant differences were considered when *P* < 0.05.

**Figure 7:**
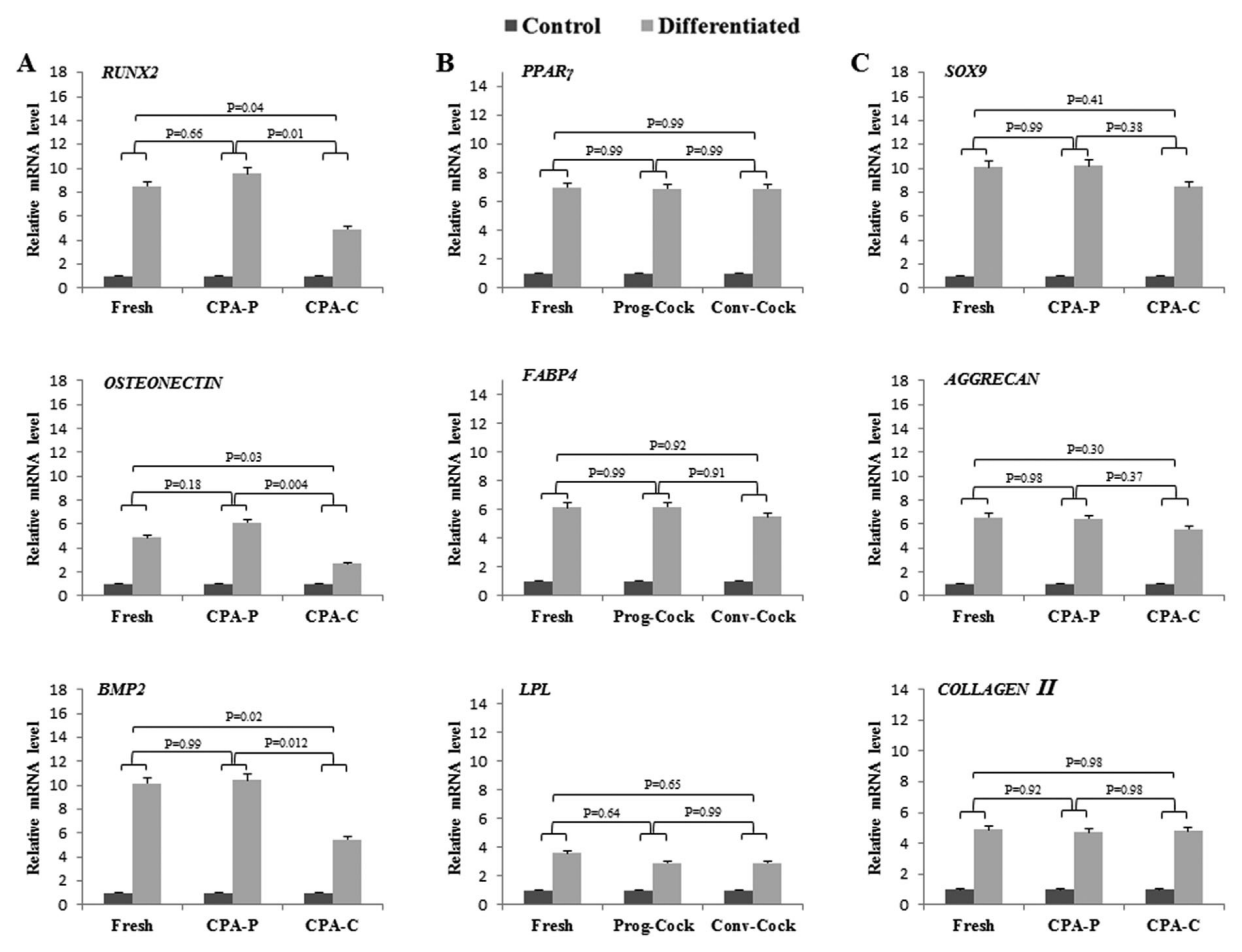
RT‐PCR analysis in cultured WJMSCs. (A) Relative m‐RNA level of osteogenic specific marker genes. (B) Relative m‐RNA level of adipogenic specific marker genes. (C) Relative m‐RNA level of chondrogenic specific marker genes. Significant differences were considered when *P* < 0.05.

**Figure 8:**
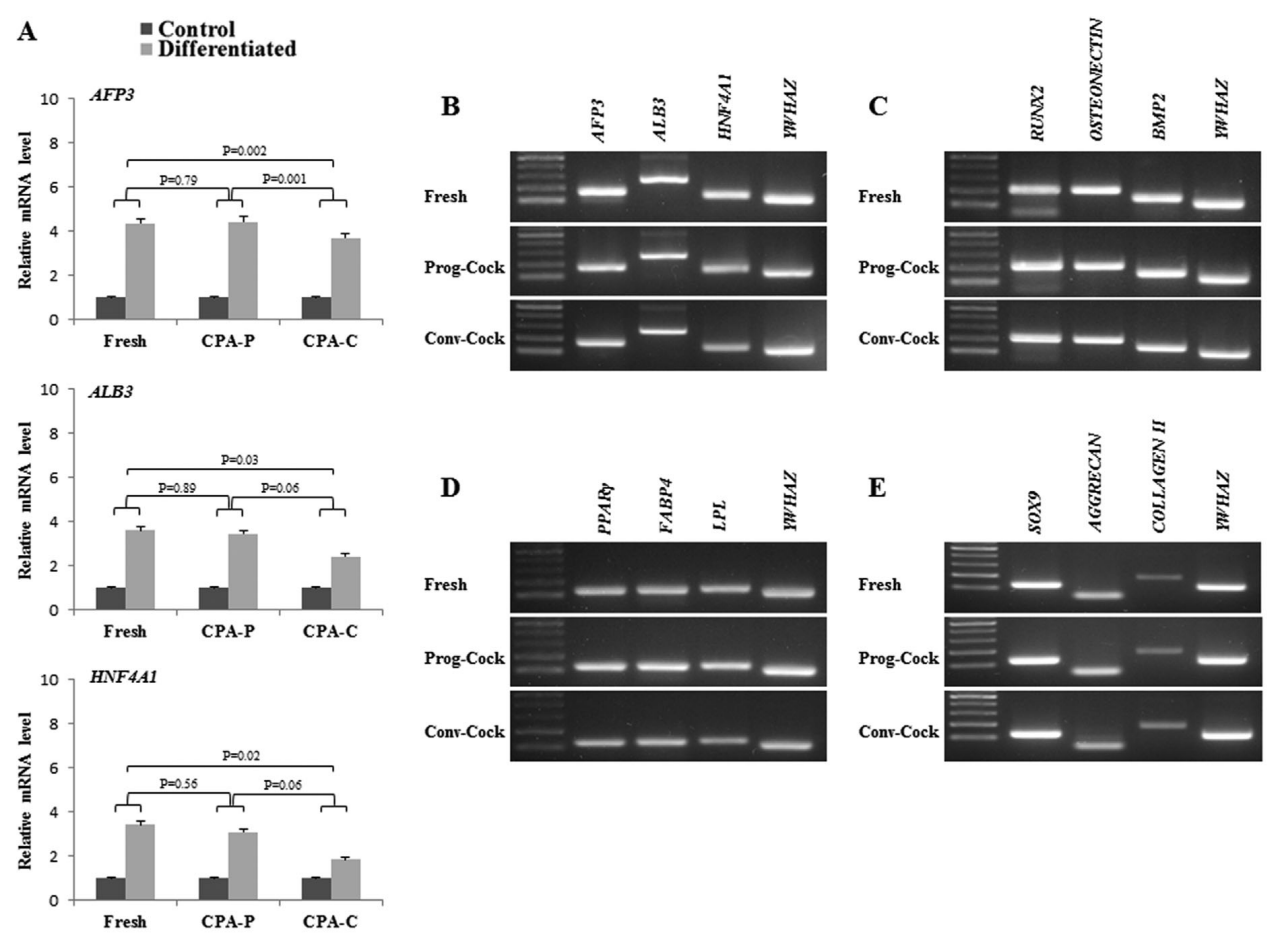
RT‐PCR analysis in differentiated hepatocyte‐like cells. (A) Relative m‐RNA level of hepatocyte specific marker genes and (B) their product size. (C–E) Represents the product sizes of Figure 7 (A–C). Significant differences were considered when *P* < 0.05.

Total RNA and protein were isolated from WJMSCs at passage 3 in all the experimental groups. There was no significant change observed in the expression of transcription factors such as *OCT4*, *SOX2*, and *NANOG* among Fresh and cryopreserved groups (Fig. 6A and C). The expression of pro‐apoptotic factors such as *BAX*, *p53*, and *p21* was significantly (*P* < 0.05) elevated, whereas the expression of anti‐apoptotic factor *BCL2* was significantly (*P* < 0.05) reduced in WJMSCs isolated from Conv‐Cock group compared to that in WJMSCs of Fresh and Prog‐Cock group (Fig. 6B and D). The *BAX*/*BCL2* ratio was found to be 1.35 ± 0.36% and 2.26 ± 0.54% in WJMSCs isolated from the Prog‐Cock and Conv‐Cock group, respectively. Similar expression patterns were observed in western blot at the protein level for both transcription‐ and apoptosis‐related factors (Fig. 5C). The mRNA levels of osteocyte, adipocyte, chondrocyte, and hepatocyte lineage specific markers were found to be significantly (*P* < 0.05) increased to about 2.6‐ to 10.4‐fold, 2.9‐ to 6.9‐fold, 4.7‐ to 10.2‐fold, and 1.8‐ to 4.4‐fold, respectively after differentiation in all the experimental groups (Figs. 7 and 8). There was no significant (*P* < 0.05) differences were observed in the expression of adipocyte and chondrocyte lineage specific markers among all the experimental groups. However, the WJMSCs isolated from Conv‐Cock group showed significantly (*P* < 0.05) reduced expression of osteocyte and hepatocyte lineage specific markers after differentiation compared to WJMSCs under Fresh and Prog‐Cock group (Figs. 7 and 8).

## DISCUSSION

Wharton's jelly is a promising tissue source of MSCs for both autologous and allogeneic applications. Although several studies have reported the successful cryopreservation of WJMSCs, cryopreservation of Wharton's jelly tissue as a whole instead of WJMSCs possesses several advantages as described in the Introduction. DMSO is the most commonly used cryoprotectant along with FBS. However, the use of these two components in cryosolution impedes the clinical utility of MSCs. In this study, we demonstrate that the Wharton's jelly tissue can be cryopreserved using DMSO‐ and serum‐free cocktail cryosolution comprising of 0.05 M glucose, 0.05 M sucrose, and 1.5 M ethylene glycol in PBS in conjunction with programmed slow freezing method (Prog‐Cock) developed in our laboratory.

In this study, the minimum cell survivability was observed when 10% DMSO supplemented with 10% FBS was used as CPA compared to the cocktail solution (Cock). The reason for this reduction in cell survivability could be multifactorial, but the general rationale is that during cryopreservation, structured multicellular tissues, and simple cell suspensions may respond differently to cryoprotective agents (CPA), cooling, warming, and dehydration. Tissues are generally known to be impervious to cold shock due to their structural complexity compared to single cells. However, the use of DMSO as CPA may increase the sensitivity of certain tissues to cold shock upon cooling before freezing [Morris et al., 1983; Morris, 1987]. Further, the requirement of optimal CPA concentration may also vary from simple cell suspensions to complex tissues. Therefore, typically employed concentrations of DMSO (8–20%) for cell preservation may not be sufficient for tissue preservation, since this concentration is not sufficient to penetrate adequately deep into the tissues to limit intracellular ice formation. Based on these previous observations, we speculate that Wharton's jelly tissue might have undergone cold shock when DMSO is used as CPA or it may require higher DMSO concentration than currently used in this study. Further, we cannot completely exclude the possibility that the remnant DMSO in freeze‐thaw WJ tissue even after washing might have exerted its toxicity resulting in poor cell viability. Therefore, washing step plays an important role in removing DMSO from these complex tissues and requires additional washing procedures. The deleterious effect of DMSO due to cold shock may possibly be circumvented by using combinations of two or more cryoprotectants resulting in an additive or synergistic enhancement of cell survival while reducing cytotoxicity. Although it has been demonstrated that increasing DMSO concentration to 6 M can result in higher cell survivability of porcine articular cartilage [Jomha et al., 2004], this higher DMSO concentration may exert considerable cytotoxicity and may not be a better option for clinical utility. Therefore, in the present study, we have not attempted to either increase the DMSO concentration or supplementing DMSO with other CPAs. Instead, a cocktail solution with a combination of 0.05 M glucose, 0.05 M sucrose, and 1.5 M ethylene glycol in PBS was used as CPA, which we have earlier used in our laboratory for cryopreservation of human dental follicle tissue. The rate of cell survivability in cryopreserved WJ tissue with the cocktail solution was similar to our earlier report [Park et al., 2014] albeit with minor variations. Previously, it has been reported that cryosolution containing both non‐permeable and permeable CPAs seem to be more advantageous than solutions containing only permeable CPAs [Shaw et al., 2000]. The present study also demonstrated that cocktail solution has greatly enhanced the effect of WJ tissue protection during cryopreservation through a possible synergistic mechanism. It is possible that the permeable CPA like ethylene glycol might have protected the cells against freezing injury by reducing ice formation inside and outside the cells whereas non‐permeable CPAs like glucose and sucrose might have dehydrated cells and extracellular matrix and thus reduced the amount of water present before freezing. In addition, glucose and sucrose might have also contributed to the stabilization of cellular membranes and proteins during freezing and drying.

In general, the biological systems are greatly influenced by cooling rate during cryopreservation. Each system tends to have its own specific optimal cooling rate, with decreased survival at cooling rates that are too low (slow‐cooling damage) or too high (fast‐cooling damage) [Mazur et al., 1972]. At very slow cooling rates, the cryoinjury occurs due to the solution effects (i.e., the solute concentration and severe cell dehydration) whereas, at high cooling rates, cryoinjury occurs due to the lethal intracellular ice formation. So the optimal cooling rate falls in a range that is neither too fast nor too slow. In this context, the programmed freezing protocol may provide an improved cryoprotection for cells and tissues. Therefore, we further compared a programmed freezing protocol, previously optimized in our laboratory for cryopreservation of dental follicle tissue, with a conventional method (−1°C/min). The present study demonstrated improved cell survivability in programmed freezing method compared to the conventional method for both the CPAs. This improved cell survivability during programmed freezing may be due to the factors such as hold‐time and plunging temperature. A suitable hold‐time is required for any cryopreservation protocol, as cryoprotectant cannot osmose into cell membrane with shorter hold‐time or it may exert chemical toxicity to cells with longer hold‐time. Therefore, a suitable hold‐time allows the cryoprotectant to osmose into cell membrane without exposing them to the cryoprotectant for too long time.

As this study mainly focused on the use of DMSO‐ and serum‐free cryosolution, and also due to the reduced WJMSCs survivability noted in WJ tissue cryopreserved with DMSO, only WJMSCs isolated from WJ tissue cryopreserved with cocktail solution (Conv‐Cock and Prog‐Cock) were further characterized to evaluate the effect of cryopreservation on their basic stem cell characteristics. Most of the studies report maintenance of morphological and functional characteristics of stem cells even after cryopreservation. However, safe cryopreservation of stem cells and its efficacy for clinical utility depends on several factors such as freezing temperature, freezing rate, freezing duration, cryoprotectant used, thawing, and removal of cryoprotectant. Nevertheless, the success of any stem cell therapy often depends on repeated transplantations and, therefore, relies on freezing and storage of cells. For instance, in patients with chronic heart failure or ischemic heart disease, chronically failing hearts with no recent infarct may not respond to MSC for the first time upon injecting cells into the ischemic area and thus, more than one injection could be necessary to obtain better results [Lee et al., 2004]. Therefore, it is crucial to evaluate the possible effect of any freezing protocol on the change in MSC phenotype and functional characteristics. In this study, colonies of adherent and fibroblastic spindle‐like morphology of WJMSCs were observed on day 5 from both fresh and cryopreserved WJ tissue. However, it is possible to notice that the decrease in cell recovery and altered biological characteristics of WJMSCs was directly related to the freezing rate, since WJMSCs from WJ tissue stored using conventional method (−1°C/min) showed a retarded post‐thaw cell growth and reduced cell clumps on day 5 resulting in lower cell recovery in primary culture. Moreover, in these cells, the effect of possible freezing injury seemed to be irreversible even after in vitro propagation as indicated by reduced colony forming ability with prolonged doubling time. Although we did not find any significant post‐thaw morphological changes among cryopreserved groups compare to the fresh group, earlier it has been reported that cryopreservation‐induced morphological changes such as extensive branching of cytoplasmic extensions may affect the proportion of viable cells reattaching on culture dishes [Heng, 2009]. This suggests that programmed freezing method may preserve the better ability of post‐thaw cells to maintain higher colony forming ability. Therefore, the conventional method of WJ tissue freezing with cocktail cryosolution (Cock) may require long‐term post‐thaw in vitro propagation to get adequate cell doses for clinical utility. But this further enhances the risk of culture induced epigenetic changes as well as bacterial and viral contaminations.

According to the guidelines of The International Society for Cellular Therapy (ISCT), mesenchymal stem cells should express CD105, CD73, and CD90; and lack the expression of CD45 and CD34; CD14 or CD11b; CD79alpha or CD19; and HLA‐DR surface molecules, adhere to a plastic surface when maintained under standard culture conditions and must differentiate to osteoblasts, adipocytes, and chondroblasts in vitro [Dominici et al., 2006]. In our study, WJMSCs retained their expression of CD markers even after cryopreservation in both conventional and programmed freezing method, implying that freezing rate had no effect on the integrity of cells. But the number of cells expressing CD73 was significantly reduced after conventional freezing. CD73 has been reported to play an important role in osteoblast differentiation [Takedachi et al., 2012]. The present study also indicated the diminished expression of CD73 after conventional freezing might have reduced the propensity of WJMSCs towards osteoblast differentiation while maintaining adipogenic and chondrogenic differentiation ability as indicated by lineage‐specific m‐RNA marker expression. Further, conventional freezing of WJ tissue using cocktail solution has compromised transdifferentiation ability of WJMSCs into hepatocyte‐like cells compared to the programmed freezing method. In the present study, the expression of early transcription factors such as *OCT4*, *SOX2*, and *NANOG* was not affected by cryopreservation of WJ tissue using both conventional and programmed freezing method. These transcription‐related proteins were mainly localized to the nucleus while NANOG was occasionally detected in the cell cytoplasm in all the experimental groups and these results are in agreement with the previous report [Carlin et al., 2006]. However, it is not clear whether this subpopulation of cytoplasmic NANOG‐positive cells will increase the risk of in vivo tumorigenicity or not, since MSCs found in the cervical cancer stroma display cytoplasmic NANOG expression and can promote the progression of cervical cancer in vitro and in vivo [Gu et al., 2012].

The major limitation for using any freeze‐thaw tissues for clinical utility is attaining adequate viable cell numbers. A significant number of cells lose their viability during freezing and thawing procedures as a result of cryopreservation‐induced apoptosis [Schmidt‐Mende et al., 2000]. However, immediate post‐thaw cell viability cannot be a true measure of representing the efficacy of cryopreservation. Therefore, we further evaluated the possible recurrence of apoptosis due to cryoinjury in post‐thaw cultured cells using Annexin V‐FITC assay. The results showed significantly greater apoptosis signals for conventional method than the programmed method of freezing. However, we found a small proportion of cells undergoing apoptosis in WJMSCs isolated from fresh WJ tissue. This could be due to the effect of passaging since the assay was conducted in passage 1 cells. Further, an elevated expression of apoptotic‐related factors both at m‐RNA and protein level in WJMSCs isolated from WJ tissue frozen using conventional method suggesting the possible occurrence of apoptosis due to cryoinjury resulting in loss of post‐thaw cell survivability. The present study also speculates the possible occurrence of DNA damage in WJMSCs isolated from WJ tissue stored using conventional method due to cryoinjury, because of higher expression of p53 and p21 both at m‐RNA and protein level with a higher proportion of cells in the G_0_/G_1_ phase of cell cycle.

In conclusion, the cocktail solution (Cock) comprising of 0.05 M glucose, 0.05 M sucrose, and 1.5 M ethylene glycol in PBS showed higher post‐thaw cell survivability in conjunction with the programmed method of freezing. This study also indicated the typical concentration of DMSO (8–20%) used for preservation of simple cell suspensions may not be sufficient for complex tissue preservation. Nevertheless, the feasibility of developing cocktail cryosolutions with both permeating and non‐permeating CPAs could synergistically increase the cryoprotection while reducing or completely eliminating the use of cytotoxic DMSO and xenogeneic serum components. Poor cell recovery, growth characteristics, apoptosis, and loss of basic stem cell characteristics noted in conventional freezing method suggested that freezing rate also plays an important role in tissue cryopreservation apart from cryoprotectants used. Although present study has demonstrated the feasibility of using DMSO‐ and serum‐free cryosolution for short‐term WJ tissue banking with controlled rate freezing in vitro, further studies are needed to evaluate the effect of this cryosolution on MSCs stored for longer periods of time with in vivo efficacy of post‐thaw cells.
